# RATS切肺有优势，但不会止步于切肺

**DOI:** 10.3779/j.issn.1009-3419.2018.03.19

**Published:** 2018-03-20

**Authors:** 振涛 于

**Affiliations:** 天津医科大学肿瘤医院胸外科 Department of Thoracic Surgery, Tianjin Tumor Hospital Affiliated to Tianjin Medical University

自从2000年达芬奇机器人手术（robot-assisted thoracic surgery, RATS）系统获得了美国食品药品监督管理局（Food and Drug Administration, FDA）批准并大量应用于临床各个领域，微创外科历史掀开了崭新的一页^[1]^。虽然，RATS治疗各种胸部疾患是否较VATS具有一定的优势仍存在广泛的争议，但是，今年来随着国内达芬奇机器人的广泛引入，越来越多的胸外科医生已经将它用于肺癌、食管癌及纵隔肿瘤的外科治疗中。目前已开展RATS肺叶切除术、RATS解剖性肺段切除、RATS支气管袖式切除、食管良性肿瘤的剥除术、食管癌根治术（包括胸内吻合）以及全胸腺切除术等。

上海肺科医院姜格宁教授撰文总结RATS优势在于：三维视野下定位精确，手术姿态稳定；符合人体工学的手术器械，器械再定位容易，过滤手颤抖，移动度缩小，可完成精确的缝合，结扎；其缺点包括：术中缺乏大局观和触觉感，费用高，设备安装耗时等^[2]^。

王述民教授的团队是我国较早开展RATS肺癌手术的单位之一，也是世界最早完成千例RATS胸部手术的专家之一，多年来他们总结了丰富的临床经验，为推动RATS胸外科手术的发展做出了巨大的贡献。在本文中，作者回顾性分析自2014年1月-2017年1月行达芬奇机器人手术以及行电视胸腔镜辅助手术治疗非小细胞肺癌患者的相关临床资料，两组各纳入45例患者并进行配对的对照研究。结果显示两组均无中转开胸及围术期死亡。机器人组与胸腔镜组的术中失血量[（50.30±32.33）mL *vs*（208.60±132.63）mL]与术后第1日胸引液的量[（275.00±145.42）mL *vs*（347.60±125.80）mL]比较，机器人组均少于胸腔镜组；机器人组与胸腔镜组的淋巴结清扫数量[（22.67±9.67）个*vs*（15.51±5.41）个]及淋巴结清扫站数（6.31±1.43）站*vs*（4.91±1.04）站]比较，机器人组均多于胸腔镜组，差异具有统计学意义；两组的其他指标比较，差异均无统计学意义。他们认为达芬奇机器人行非小细胞肺癌根治术安全、有效，在近期术后疗效上优于胸腔镜组。

这样的结论也符合现今已有的多数研究的结果。许多的研究表明，在术中出血量、术后并发症（尤其是肺部并发症）、术后引流量、麻醉药物的使用时间和剂量、住院时间等方面，RATS肺癌手术均有一定的优势。2014年Farivar等^[3]^分析了STS数据库，显示RATS手术患者，住院时间缩短、30天死亡率降低。

然而，Swanson等^[4]^也提出不同的结论，机器人切除肺叶和楔形切除术的费用高，手术时间长，与VATS手术相比不良事件无明显差异。2014年Paul等^[5]^的研究也表明，与VATS相比，机器人手术有较高的术中损伤和出血率（机器人5% *vs* VATS 2%），费用较高。Louie等^[6]^分析2016年STS数据库后报道，在Ⅰ期和Ⅱ期患者中，机器人肺叶切除手术带来更多的并发症而且手术时间明显延长，这或许也与日益提高的VATS手术水平有关。

2016年Cerfolio等^[7]^报道632例机器人手术病例中仅有15例（2.4%）发生血管相关并发症，他认为在机器人手术中可以有效防止和处理血管损伤。

对于RATS手术的长期生存情况，近年来也有一些相关报道。2012年Park等^[8]^一项针对325例RATS手术患者的多中心研究中报告，5年生存率为80%（Ⅰa期91%、Ⅰb期83%、Ⅱ期49%），这是比较好的一组结果，表明机器人胸外科手术安全有效，其5年生存率与其他手术相似。2017年的一项比较开放切开术，胸腔镜手术和机器人手术等的研究报道，两种微创方法的临床Ⅰ期非小细胞肺癌的治疗结果与开胸肺叶切除术有相似的长期生存率，而VATS和机器人技术与缩短住院时间有关，而机器人手术带来了更多的淋巴结清扫数量^[9]^。

达芬奇机器人食管癌手术（robot-assisted minimally invasive esophagectomy, RAMIE）在食管癌微创治疗尚未得到普遍接受的情形下，其临床应用也日益受到关注。2015年荷兰医生Ruurda^[10]^在*Journal of Surgical Oncology*发表综述文章，检索了已发表的符合分析方法的文章仅16篇，只有约300例报告，反映了机器人手术在早期阶段的实际状况。手术路径同其他开胸手术一样，包括两步骤手术（胸内吻合）或三步骤手术（颈部吻合）。手术体位同食管VATS手术，主要采用两种：俯卧位和左俯卧位/侧卧位。戳卡的位置略有不同；有些术者使用额外的机械臂（第三臂）。手术过程的失血从75 mL-640 mL，中转开胸比例从0到21%，住院时间（7天-21天），ICU住院时间（0.5天-3.5天），均在正常范围。大多数文献都报道了并发症。最常见的是肺炎，6%-45%，吻合口狭窄（10%-68%），心脏并发症（通常是房颤，5%-36%）；如果是颈部吻合，喉返神经损伤（4%-35%）。吻合口瘘在各组报道中有所不同，4%-38%，术后死亡率很低（0.5%-6%）。预后结果并非均有报道，但根治性切除率81%-100%、淋巴结清扫数目为18个-38个之间。

RATS胸部手术发展至今逐渐被我国的部分胸外科医生接受，并以极快的速度积累着病例和临床经验，无论在食管外科、肺外科，还是纵隔外科方面，我们无论在微创技术基础上，还是在病例数量上都处于世界前列，也希望更多的同仁能够同心聚力、协作发展，带给学术界更多的中国经验、中国规范，让这个新兴的外科领域成为我们的天地！
